# Kimura disease as an uncommon cause of persistent hypereosinophilia: a diagnostic challenge

**DOI:** 10.11613/BM.2023.020801

**Published:** 2023-04-15

**Authors:** Javier Laguna, María Rodríguez-García, Angel Molina, Anna Merino

**Affiliations:** Biochemistry and Molecular Genetics Department, Core Laboratory, Biomedical Diagnostic Centre, Hospital Clínic, Barcelona, Spain

**Keywords:** eosinophils, hematology, kimura disease, leukocyte disorders

## Abstract

Kimura disease (KD) is an unusual inflammatory disease of unknown etiology. Despite being described many years ago, KD might cause diagnostic difficulty or be confused with other conditions. Here, we present a 33-year-old Filipino woman who was referred to our hospital for evaluation of persistent eosinophilia and intense pruritus. Blood analysis and peripheral blood smear review showed high eosinophil counts (3.8 x10^9^/L, 40%) that did not show morphological abnormalities. Besides, high serum IgE concentration was detected (33,528 kU/L). Serological tests were positive for *Toxocara canis* and treatment with albendazol was initiated. Nevertheless, increased eosinophil counts were still present after several months, alongside with high serum IgE concentrations and intense pruritus. During her follow-up, an inguinal adenopathy was detected. The biopsy revealed lymphoid hyperplasia with reactive germinal centers and massive eosinophil infiltration. Proteinaceous deposits of eosinophilic material were also observed. All these findings, together with peripheral blood eosinophilia and high IgE concentrations, confirmed the diagnosis of KD. The diagnosis of KD should be considered in the differential diagnosis of long-standing unexplained eosinophilia in association with high IgE concentrations, pruritus and lymphadenopathies.

## Introduction

Kimura disease (KD) is a rare lymphoproliferative inflammatory disease of unknown etiology. This disease was first described in 1937 as “eosinophilic hyperplastic lymphogranuloma” (*1*). However, in 1948, it was renamed “Kimura disease” after the publication by Kimura *et al*., in which the clinicopathological characteristics of this entity were described (*2*).

The typical presentation of KD is pruritus and painless subcutaneous mass lesions, predominantly on the head and neck, often with regional lymphadenopathy or salivary gland involvement. Nonetheless, it has been reported that these mass lesions can appear in other parts of the body, particularly visceral involvement. Kimura disease can be systemic and involve multiple organs. Kidney involvement is the main systemic manifestation, associated to proteinuria and nephrotic syndrome (*3*, *4*). Considering laboratory results, the most frequent findings are increased eosinophil counts in peripheral blood and high serum immunoglobulin E (IgE) concentration.

Despite being described more than 70 years ago, KD is a disease that can be difficult to diagnose. Other malignancies such as T-cell lymphoma and Hodgkin lymphoma are included in the differential diagnosis along with other benign diseases as allergic reaction or parasitic infection. For this reason, we describe a new case of KD with the aim of sharing information and contributing to an early diagnosis of this disease. In addition, we studied the frequency of KD among severe eosinophilias observed in our laboratory over a one-year period.

## Case report

Written informed consent was obtained from the patient. A 33-year-old Filipino woman, living in Spain for a year, was referred to our hospital for evaluation of eosinophilia in December 2017. High eosinophil counts had been observed in October and November 2017. She suffered intense pruritus and skin lesions due to scratching. Physical examination did not reveal adenopathies or organomegalies.

Samples were collected in 5 mL BD Vacutainer tubes (BD Vacutainer Systems, Plymouth, UK) with clot activator and gel for serum separation (for biochemical measurements) and 5 mL BD Vacutainer tubes with EDTA (for haematological tests). Complete blood count (CBC) and biochemical parameters were analysed on the ADVIA 2120i and Atellica Solution, respectively (both analysers from Siemens Healthineers, Tarrytown, USA). The proteinogram was performed by capillary electrophoresis using CAPILLARYS 2 (Sebia, Lisses, France).

**Figure 1:**
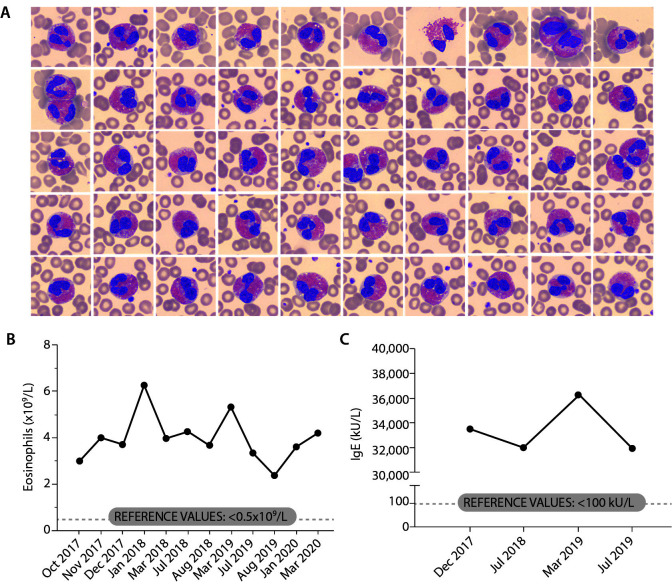
A) High number of eosinophils without morphological abnormalities observed in the peripheral blood review. Staining with May Grünwald-Giemsa. Images obtained in CellaVision DM96 (1000x). B) Evolution of eosinophil count in peripheral blood during 2017-2020. C) Evolution of serum IgE concentration during 2017-2019.

An overview of the laboratory findings is shown in Table 1. All parameters were measured in samples collected in December 2017. Complete blood count showed leukocytes, haemoglobin and platelet values within the reference range. Nevertheless, eosinophil counts were high (3.8 x10^9^/L, 40% of total white blood cell count). All other haematological parameters were normal. Regarding biochemical parameters, the serum total protein concentration was slightly elevated, with a high proportion of γ-globulins. To confirm the eosinophilia observed on the ADVIA 2120i analyser, a peripheral blood smear review was performed using CellaVision DM96 (CellaVision, Lund, Sweden). Eosinophilia was observed, and eosinophils showed no morphological abnormalities (Figure 1A).

**Table 1:** Relevant laboratory test results obtained in the 33-year-old female patient with Kimura disease (December 2017)

	**Result**	**Reference values**
**Complete blood count**		
RBC (x10^12^/L)	4.49	3.90-5.50
Haemoglobin (g/L)	136	120-170
WBC (x10^9^/L)	9.4	4.0-11.0
Eosinophils (x10^9^/L)	3.8*	< 0.5
Platelets (x10^9^/L)	207	130-400
**Serum biochemical parameters**		
Glucose (mmol/L)	4.6	3.6-6.1
Creatinine (μmol/L)	51	26-115
GFR (mL/min/1.73m^2^)	90	> 90
LD (U/L)	436	250-450
Total protein (g/L)	85*	63-80
Proteinogram: Albumin (%) α1-globulins (%) α2-globulins (%) β-globulins (%) γ-globulins (%)	45.6 4.2 9.2 13.0 28.0*	55.8-66.1 2.9-4.9 7.1-11.8 8.4-13.1 11.1-18.8
**Urine biochemical parameters**		
Total protein (mg/L)	91	< 100
**Complementary tests for eosinophilia evaluation**		
Vitamin B12 (pmol/L)	308	> 220
Tryptase (mg/L)	4	< 13
CRP (mg/L)	0.2	< 10
Serum IgE (kU/L)	33,528*	< 100
*abnormal values. CRP - C-reactive protein. GFR - glomerular filtration rate. IgE - immunoglobulin E. LD - lactate dehydrogenase. RBC - red blood cells. WBC - white blood cells.		

When eosinophilia was confirmed, laboratory evaluation was completed according to the latest recommendations (*5*). Vitamin B12, tryptase, C-reactive protein (CRP) and IgE were measured. Vitamin B12 and CRP were measured in the Atellica Solution. Tryptase and IgE were measured using the ImmunoCAP (ThermoFisher, Waltham, USA). All test results were within the reference range, except for the extremely high IgE values (33,528 kU/L). In addition, measurement of IgG antibodies activity to *Trichinella spiralis*, *Taenia solium*, *Strongyloides stercoralis* (Scimedx Corporation, Denville, USA) and *Toxocara canis* (R-Biopharm, Darmstadt, Germany), measurement of total antibodies to *Fasciola hepatica* and *Echinococcus granulosus* (Laboratoires Fumouze, Levallois-Perret, France), and study of microfilariae in blood and parasites in faeces (protozoa, helminths) by optical microscopy were performed, showing positivity for *Toxocara canis* (antibody titers: 5.29; threshold for positive results > 1.1). Therefore, *Toxocara canis* infection was thought to be the cause of eosinophilia and treatment with albendazole was started.

After seven months (July 2018), pruritus persisted along with increased eosinophil values in peripheral blood and high serum IgE concentrations, as shown in Figures 1B and 1C, respectively. The microbiological studies described in the previous paragraph were repeated and all were negative. As the cause of the eosinophilia was still not found, bone marrow aspirate was performed for the study of myeloproliferative diseases (August 2018). Bone marrow aspirate showed cells without morphological abnormalities, eosinophilia, lymphoplasmacytosis and absence of blast cells. Karyotype and *BCR*-*ABL* and *FIP1L1*-*PDGFRA* fusion gene studies by RT-PCR were performed on this bone marrow sample, and all were negative. An immunophenotypic study by flow cytometry was also performed, showing a slight increase in the B-lymphocyte population. Since the cause of the eosinophilia was not determined, patient follow-up and monitoring of eosinophilia over time were recommended.

The patient was undiagnosed until March 2020, when an inguinal adenopathy was detected during a physical examination. A computerized tomography scan confirmed enlarged axillary, inguinal and iliac lymphadenopathies. Inguinal lymphadenopathy biopsy was performed in May 2020. Histopathological examination showed lymphoid hyperplasia with reactive germinal centres and massive infiltration by eosinophils. In addition, proteinaceous deposits of eosinophilic material were observed in germinal centres. Eosinophilic microabscesses were also present. All these findings, together with peripheral blood eosinophilia and high IgE concentrations, confirmed the definitive diagnosis of KD.

Between May 2020 and July 2022, the patient stopped attending the hospital. Treatment with Xolair (omalizumab) was started in July 2022. Omalizumab is a monoclonal antibody that binds specifically to IgE, and it is administered subcutaneously. So far, a good response has been observed, with a decrease in pruritus and eosinophil count (currently 0.6 x10^9^/L).

### Severe eosinophilia cases detected in our laboratory during 2019

**Figure 2:**
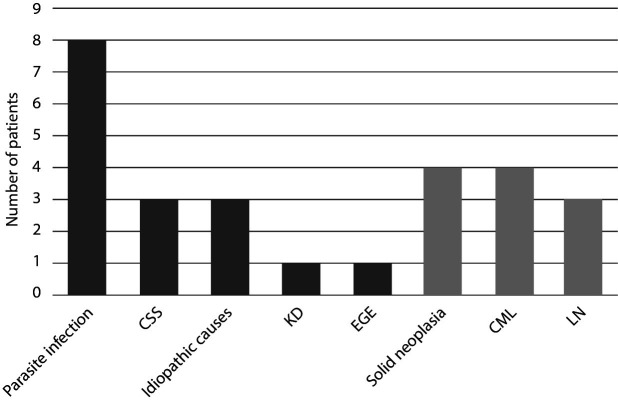
Diagnosis of patients with severe eosinophilia (above 5 x10^9^/L): non-malignant diseases (in black) and solid neoplasia and haematological diseases (in grey). CML - chronic myeloid leukaemia. CSS - Churg-Strauss syndrome. EGE - eosinophilic gastroenteritis. KD - Kimura disease. LN - lymphoid neoplasm.

In our hospital, we performed more than 330,000 CBC in 2019. However, only 3% of the samples had eosinophil counts above 0.5 x10^9^/L, being mild eosinophilia (up to 1.5 x10^9^/L) the most common (92.8%). Moderate (between 1.5 and 5 x10^9^/L) and severe eosinophilia (above 5 x10^9^/L) were less frequent (6.5% and 0.7%, respectively). The 72 samples with severe eosinophilia belonged to 27 patients (Figure 2). The most common causes of severe eosinophilia are non-malignant diseases. Among these patients, we observed our patient reported in this case, as she presented eosinophil counts above 5 x10^9^/L during her follow-up.

## Discussion

This case is an example of a low frequent cause of hypereosinophilia that can go unnoticed or be misdiagnosed by clinicians. In the case presented herein, the time elapsed from the detection of eosinophilia to the definitive diagnosis was 30 months. We highlight the importance of taking this disease into account in the differential diagnosis of eosinophilia, since treatment can benefit the patient with a decrease in the number of circulating eosinophils in peripheral blood and an improvement in their symptoms.

The study of eosinophilia is difficult, because it is an uncommon phenomenon and is not a specific finding of a single disorder. Recently, Larsen and Savage published a review about the differential diagnosis of eosinophilia according to the 2016 WHO classification of eosinophilic disorders (*5*, *6*). Due to the fact that eosinophilia is secondary (or reactive) in most cases, these causes must be discarded in first place. Many causes have been described, such as parasite infection, allergic disorders, dermatological disorders, drugs, rheumatologic diseases, thrombotic diseases, gastrointestinal disorders or Churg-Strauss syndrome, among others. Kimura disease should be studied in the differential diagnosis of secondary causes of eosinophilia. In this way, bone marrow analysis, cytogenetic tests and the fusion genes study would be avoided.

It is recommended that the laboratory evaluation including serum tryptase activity, CRP, and vitamin B12 in the study of eosinophilia, because these parameters may be altered in some diseases with peripheral blood eosinophilia (*5*). Serum tryptase may be elevated in cases of anaphylactic reactions and systemic mastocytosis, vitamin B12 may be elevated in patients with chronic myeloproliferative neoplasms, and CRP may be elevated in certain rheumatic diseases (*7*-*9*). Regarding the physical examination of the patient, it is very important to assess the presence of lymphadenopathy and enlarged spleen or liver. If necessary, organ damage should be assessed by chest X-ray, echocardiogram or biopsy.

Our patient had all the typical features of KD and she did not present any systemic manifestation. It has been reported that kidney failure may be observed at the same time or long after the appearance of the masses. Proteinuria is seen in 12-16% of patients, of whom 60-80% have nephrotic syndrome (*3*). Membranous glomerulonephritis is the commonest histopathological pattern, but other patterns may be observed: mesangial proliferative glomerulonephritis, minimal-change disease, focal-segmental hyalinosis, IgM nephropathy, and IgA nephropathy (*3*, *4*, *10*). Kidney examination was performed in our patient by Doppler ultrasound scan, but kidney abnormalities were not found. Besides, she had serum creatinine and urine protein concentrations within the reference ranges.

Kimura disease is endemic in Asia, especially China and Japan. To date, Zhang *et al.* have published the longest case series (46 cases) (*3*). They observed that KD is more frequent in males in the second and third decades of life. They also studied the location of the adenopathies, and observed that the most frequent locations were the head and neck (63%). Only 13% of patients had inguinal adenopathies. The most relevant issue of our case report is the fact that the patient with KD was a 33-year-old female, being the first Filipino patient with inguinal, axillary and iliac adenopathies as a form of presentation. To our knowledge, there are only five published cases of KD in Filipino patients, and none of them had this type of adenopathies. The reported Filipino patients showed the following clinical findings: periauricular mass and cervical adenopathies in two patients, parotid mass and periorbital inflammation in two other patients and left upper eyelid inflammation and left lacrimal gland mass in one patient (*11*-*15*).

Definitive diagnosis of KD is made by histopathological examination. Necrosis, proteinaceous deposits, vascularization of germinal centres and eosinophilic microabscesses are present (*3*, *4*, *16*).

Despite being a benign disease, recurrences in KD are very common, reported in up to 60 to 80% (*4*). It is interesting to note that a peripheral T-cell lymphoma was diagnosed in three patients in the follow-up (*3*, *17*, *18*). In two of the three cases, the lymphoma was diagnosed shortly after the diagnosis of KD (one and two years later). In the third case, the lymphoma appeared 38 years later. For this reason, the clonal analysis of T-cell subsets is useful in the follow-up. Immunophenotyping is being regularly performed in our patient and no abnormal T-cell populations have been detected until now.

There is no consensus on treatment because there are no large-scale studies. However, surgery has been considered the primary treatment for KD, and radiotherapy is used for patients with incomplete resections or recurrences. In patients who do not want to be treated by surgery, immunosuppressive medications can be used (*3*, *19*, *20*). However, in recent years a new therapeutic option based on the use of antibodies is emerging. Patients successfully treated with omalizumab, benralizumab, dupilumab and mepolizumab have been reported in the literature (*21*-*24*).

The limitation of the study is that, since the exact etiology of KD is unknown, it has not been possible to demonstrate the existence of abnormalities, for example through genetic studies.

In conclusion, the histopathological examination of the inguinal adenopathy revealed lymphoid hyperplasia with reactive germinal centers, massive eosinophil infiltration and proteinaceous deposits of eosinophilic material. These findings, together with peripheral blood eosinophilia and high IgE concentrations, confirmed the diagnosis of KD. This entity should be considered in the differential diagnosis of a long-term unexplained eosinophilia in association with elevated serum IgE, pruritus and lymphadenopathy, especially in Asian patients. As we reported in this case, performing a lymph node biopsy together with complementary laboratory tests can contribute to the definitive diagnosis.
